# Identification of degradation impurity of TGR5 receptor agonist-ZY12201 by LC–MS technique during force degradation study

**DOI:** 10.1007/s42452-021-04660-y

**Published:** 2021-05-25

**Authors:** Chandrakant Sojitra, Chintan Dholakia, Padmaja Sudhakar, Kumar K. Singh, Sameer Agarwal

**Affiliations:** 1 API Division, Cadila Healthcare Limited, Sarkhej-Bavla N.H. No. 8 A, Changodar, Ahmedabad, 382 210 India; 2 Department of Chemistry, Faculty of Science, M.S. University of Baroda, Baroda, 390 002 India; 3 Zydus Research Centre, Cadila Healthcare Ltd., Sarkhej-Bavla N.H. No. 8 A, Moraiya, Ahmedabad, 382 210 India

**Keywords:** LC–MS, TGR5 agonist, Force degradation study, Oxidized product, Stress testing

## Abstract

Forced degradation study is a systemic characterization of degradation products of active pharmaceutical ingredient (API) at conditions which posses more harsh environment that accelerates degradation of API. Forced degradation and stability studies would be useful in selection of proper, packaging material and storage conditions of the API. These are also useful to demonstrate degradation pathways and degradation products of the API and further characterisation of the degradation products using mass spectrometry. TGR5 is a G protein-coupled receptor, activation of which promotes secretion of glucagon-like peptide-1 (GLP-1) and modulates insulin secretion. The potent and orally bioavailable TGR5 agonist, ZY12201, shows activation of TGR5 which increase secretion of GLP-1 and help in lowering blood glucose level in animal models. Hence it is necessary to establish and study degradation pathway and stability of API for better handling and regulatory approval. Force degradation studies of ZY12201 have shown presence of one oxidative impurity during oxidative degradation in HPLC analysis. The oxidized product is further characterized by LC–MS to elucidate structure of impurity and characterize its degradation pathway.

**Supplementary Information:**

The online version contains supplementary material available at 10.1007/s42452-021-04660-y.

## Introduction

Type 2 diabetes mellitus (T2DM) is a metabolic disorder sparked by insulin resistance and dysfunction of the β cells. Type-2 diabetes is generally characterized by increase in the resistant to insulin which leads to higher blood glucose level [1]. Impaired insulin sensitivity caused by malfunction of production, secretion and/or transport of insulin to an insulin receptors [2]. More recently, early studies have revealed that about 25% of people who went to the hospital with severe COVID-19 infections had diabetes. Those with diabetes mellitus were more likely to have serious complications and to die from the virus. One reason is that high blood sugar weakens the immune system and makes it less able to fight off infections [3]. Many drug substances like metformin, sulfonylurea class and different glinides [4] have been used to reverse the resistance of insulin in different organs, tissues and liver itself [5]. However, numbers of patients are not able to achieve glycemic control. Hence, there is a need for exploring novel mechanism of action for the treatment of metabolic syndrome.

**Fig. 1:**
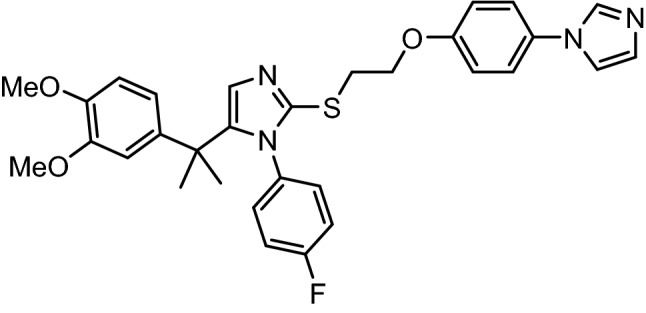
Structure of ZY12201

Bile acids plays a significance role in the emulsification lipids in the body and absorption of vitamins A, D, E, K [6]. Along with these there is strong evidence that prove function of bile acid as signaling molecules in the endocrine system which regulated various metabolic activity and indirect glucose balance [7]. TGR5 (Takeda G-protein-coupled receptor 5) is a bile acid G protein receptor which is also known as GPBAR1 [8]. We have reported the discovery of highly potent and orally efficacious TGR5 inhibitor, ZY12201 [9–11]. TGR5 receptors which is activated by bile salts are widely present in gallbladder, brain, liver, spleen and intestine [12]. TGR5 receptors are functions within tissue specific manner using increasing level of cyclic AMP present intraocular which leads to number of actions into body [13]. TGR5 receptors present at endocrine cells leads to release of glucagon like petide-1 and peptide tyrosine inhibitor that helps to hunger satiety [14, 15]. Thus, a novel TGR5 agonist may provide a treatment option for type 2 diabetes with simultaneous management of glucose levels, body weight, and associated complications. In addition, our group has also reported the effect of TGR5 agonist in combination with sitagliptin for the treatment of type-II diabetes[16]. Further we have also evaluated 2-mercapto imidazole and triazole derivatives as potential TGR5 agonist [17]. 2-((2-(4-(1H-imidazol-1-yl) phenoxy) ethyl) thio)-5-(2-(3,4-dimethoxyphenyl) propan-2-yl)-1-(4-fluoro phenyl)-1H-imidazole (ZY12201) (Fig. 1) is a potent, selective, and orally efficacious TGR5 agonist, having hTGR5 EC_50_ of 57 pM and mTGR EC_50_ of 62 pM with a favorable pharmacokinetic properties and demonstrated in-vivo glucose lowering effects in animal models (ED_50_ of 7.9 mg/kg and ED_90_ of 29.2 mg/kg) [10]. This work describes the possible degradation impurities and their characterization by force degradation study of ZY12201.

Abdelhameed et al. [18–20] have established the role of mass spectrometry in the identification and quantification of small concentration of drugs and its related impurities or metabolites. The formation of reactive metabolites during drug metabolism can result in the development of toxicities in different organs. Due to the unstable nature of reactive intermediates, capturing agents may be used which lead to the generation of stable adducts that can be extracted from the metabolic mixture and can be detected and characterized using liquid chromatography-tandem mass spectrometry (LC–MS/MS). Hence LC–MS can be proved as great tool for the identification of degradation impurities [21, 22].

## Experimental

### Chemicals and reagents

Standards and samples of ZY12201 were synthesized at Zydus Research Center, Cadila Healthcare Ltd. (Ahmedabad, India) [10, 11]. HPLC grade acetonitrile and ammonia solution and analytical grade hydrochloric acid, sodium hydroxide, ammonium acetate, hydrogen peroxide solution (30%) were purchased from Merck Specialties Pvt. Ltd. (Mumbai, India). High purity HPLC grade water was prepared by using Millipore Milli-Q plus water purification system, Bradford, PA, USA.

### Equipments and apparatus

See Table 1.

**Table 1:** Equipment/instrument details

Equipment/instrument	Manufacture
Waters HPLC–PDA system	Milford, MA, USA
Prominence Electrospray LC–MS system	Shimadzu, Japan
Analytical balance/ Micro balance	Mettler Toledo, USA
Sonicator	PCI Analytics, India
Photo stability chamber	Newtronic

### Preparation of sample solution for purity and stress studies

Test solutions of ZY12201 with 1000 µg/mL concentration were prepared in diluents and further sonicated for 5 min to dissolve the compound, which were further analyzed by HPLC.

### HPLC chromatographic conditions

A chromatography was performed on alliance waters separation module e2695 with YMC Triart C18 150 mm, 4.6 mm and 3 µm particle size column using mobile phase includes, Solvent A 10 mM ammonium acetate buffer pH (8.5 ± 0.05) and solvent B was mixture of 0.1% ammonia in acetonitrile with pump flow rate of 1.2 mL/min. The HPLC gradient mode ratios have been mentioned in Table 2. The column temperature was maintained at 35 °C. Acetonitrile and water in the ratio of 95 to 5%v/v ratio was used as a diluent. Injection volume was 15 µL. Total run time was 50 min. The retention time of ZY12201 and oxidative degraded product was 27.2 min and 18.5 min respectively. Detector wavelength was 228 nm and data processed using Empower 3 software; Version builds 3471.

**Table 2:** Gradient programme

Time	%A	%B
0.01	98	2
3	98	2
10	60	40
20	60	40
32	20	80
40	20	80
45	98	2
50	98	2

### Optimized LC–MS method parameter

An impurity obtained in the oxidation was further characterized and identified by LC–MS system. An electrospray LC–MS system (Shimadzu Prominence HPLC coupled with Triple Quadrupole Mass Spectrometer LCMS-8040 with lab solution software, version 5.72, Japan) was used for the identification of degradation impurities formed during stress testing studies. Chromatography was performed on YMC Triart C18 150 mm, 4.6 mm and 3 µm particle size column from YMC co. Ltd. Japan using mobile phase consisting of mobile phase A (10 mM ammonium acetate (pH 8.5) with ammonia solution) and mobile phase B (0.1% ammonia in acetonitrile) at a flow rate of 1.2 mL/min. The LC gradient program was applied as per Table 2. The column temperature was maintained at 40 °C. Acetonitrile—water ratio of 95:5%, v/v was used as a diluent. Injection volume was 20 µL. The analysis was carried out by using electrospray ionization mode (+ve and −ve). The capillary voltage was at 3500 V and collision energy was − 35 V. Desolvation temperature was 250 °C with nebulizing gas flow rate 180 L/h.

## Force degradation study

Forced degradation studies have been proved as an useful tool in analyzing stability of pharmaceutical products in different environmental conditions. Stability data and force degradation studies are very crucial for necessary regulatory aprovals [23]. Even ICH mandates to provide force degradation studies of new drug products along with information about potential degradants from force degradation [24, 25]. As per ICH guidelines force degradation studies should be performed under different conditions of pH, light, oxidation, dry heat, acidic, basic, hydrolysis etc. [26–28]. The FDA and ICH guidance mandate the requirement of forced degradation to recognize how the quality of a drug substance and drug product varies with time and different environmental factors [29, 30]. The structural variety of drugs makes it very difficult for development of common force degradation set of protocol. The stress conditions applied should be related to the product’s nature of decomposition [31]. The effects of chemical reactions can occurs with API are evaluated, mainly: hydrolysis of API can cause in increased humidity conditions, acidity or basicity and oxidation of API in the presence of reactive oxygen species, environmental oxygen or hydrogen peroxide, isomerization, hydration, dimerization are decarboxylation are performed to consider chemical stability of API. Photo stability tests are characterized by particular specificity and are an integral part of stability tests that are included in standard [30]. The selected approach must include products property along with details on its degradation under normal process of manufacturing, storage conditions and used conditions. Forced degradation factors necessarily include acid and base hydrolysis, thermal degradation, photolysis, and oxidation and may include freeze–thaw cycles and shear [32].

## Result and discussion

### Force degradation results

Chromatogram of the control sample for the reference in the degradation study has been given in Figure S-1.

#### Result of acid degradation hydrolysis

Test sample of 50 mg weighed in 50 mL volumetric flask, added 2–3 mL of diluent to dissolve followed by 1 mL of 5.0 M hydrochloric acid solution and heated at 60 °C for 120 min. Cool it at room temperature and neutralize the solution with 5.0 M sodium hydroxide solution with help of pH paper. The chromatogram obtained from the sample after acidic hydrolysis reaction of ZY12201 has shown a satisfactory separation of compound and the degradation products (Figure S-2).

#### Result of alkali degradation

Test sample of 50 mg weighed in 50 mL volumetric flask, add 2–3 mL of diluent to dissolve then add 1 mL of 5.0 M sodium hydroxide solution and heated at 60 °C for 120 min. Cool it at room temperature and neutralize the solution with 5.0 M hydrochloric acid solution with help of pH paper. The chromatogram for the alkali treated ZY12201 sample was achieved satisfactory (Figure S-3).

#### Result of oxidative degradation

Hydrogen peroxide has been used for the oxidative stress degradation. Electron transfer serves as basic mechanism for the oxidative forced degradation of drug substance [33]. ZY12201 sample was treated with 1 mL of 3% hydrogen peroxide solution and kept at room temperature for 4 h. Chromatographic separation of formed oxidized degradation product was achieved (Figure S-4).

#### Results of photo degradation

ICH guidelines have recommended the conditions protocols for photo stability studies [34]. Accordingly, ZY12201 samples were exposed to a minimum light of 1.2 million lux h and 200 Wh/m^2^ light; 300–800 nm wavelengths are commonly used to cause the photolytic degradation [35]. Free radical mechanism was proposed for photolytic degradation. Carbonyls, nitro aromatic, N-oxide, alkenes, aryl chlorides, weak C–H and O–H bonds, sulfides and polyenes are example of photosensitive groups present in pharmaceuticals [36]. ZY12201 sample was exposed for UV degradation under a photo stability chamber (Model: NLPS4SI) for 7 days. However, no significant degradation in product was observed with UV exposure (Figure S-5).

#### Result of thermal degradation

Sample was exposed at 105 °C for 24 h to study the thermal decomposition or thermolysis caused by heat [37]. HPLC chromatogram for thermal treated ZY12201 sample is shown in Figure S-6. Results of peak purity is shown in Table 3.

**Table 3:** Force degradation summary

Degradation condition	Time	Temp	Assay (%, w/w)	Degradation (%w/w)	Mass balance (% assay + % deg. products)	Remarks/observation
A control sample (untreated)	–	–	96.4	2.8	99.2	NA
HCl, 5.0 N (acid degradation)	2 h	60 °C	95.0	3.5	98.5	No significant degradation observed
NaOH, 5.0 N (base degradation)	2 h	60 °C	93.1	4.1	97.2	No significant degradation observed
Oxidation by 3.0% H2O2	2 h	25 °C	79.3	21.02	100.3	Oxidized product was formed
Thermally treated	24 h	105 °C	97.1	2.8	99.9	No significant degradation observed
UV treated (254 nm)	24 h	25 °C	96.9	2.75	99.7	No significant degradation observed

#### Summary of force degradation study

Force degradation studies have concluded that there was no significant changes have been found during the degradation study except oxidative degradation. Summary of force degradation study has been shown in Table 3.

### Peak purity results

Peak purity is a comparison of the reference standard to the API in the sample stressed by forced degradation to confirm that no impurity is eluted. Peak threshold is used as a parameter for determining peak purity in HPLC. For the acceptance of the peak purity, angle should be less than a purity threshold. Peak purity results are summarized in Table 4.

**Table 4:** Peak purity results

Degradation condition	Acid treated	Alkali treated	Oxidation treated	Photolytic treated	Thermal treated
Purity angle	3.467	3.557	0.702	1.855	1.512
Purity threshold	5.716	5.390	1.344	3.001	2.450

### Identification of impurity by LC–MS

Identification of degradation related impurities for ZY12201 was done in oxidation treated sample through LC–MS technique. Total eight impurities were formed during oxidation degradation, among all impurity in ZY12201 oxidation treated sample, one impurity was confirmed and identified through mass spectral analysis. The degraded impurities mass and retention time has been reported in Table 5 and mass spectra of all impurities are shown in Figure S-7.

**Table 5:** ZY12201 and its oxidation degraded impurities mass value

Retention time (min)	Mass value (M^+^)
13.57	284.10
19.63 (identified impurity)	575.25
21.95	417.20
22.45	593.30
25.91	536.30
27.09	508.25
27.88 (ZY12201)	559.25
28.70	575.25
31.57	522.25

The positive ion mass spectral analysis of impurity-1 was observed at 575 (M^+^) suggesting the possibility of Molecular formula C_31_H_31_FN_4_O_4_S, which confirms the theoretical molecular weight of Impurity-1.

## Oxidative degradation pathway

**Fig. 2:**
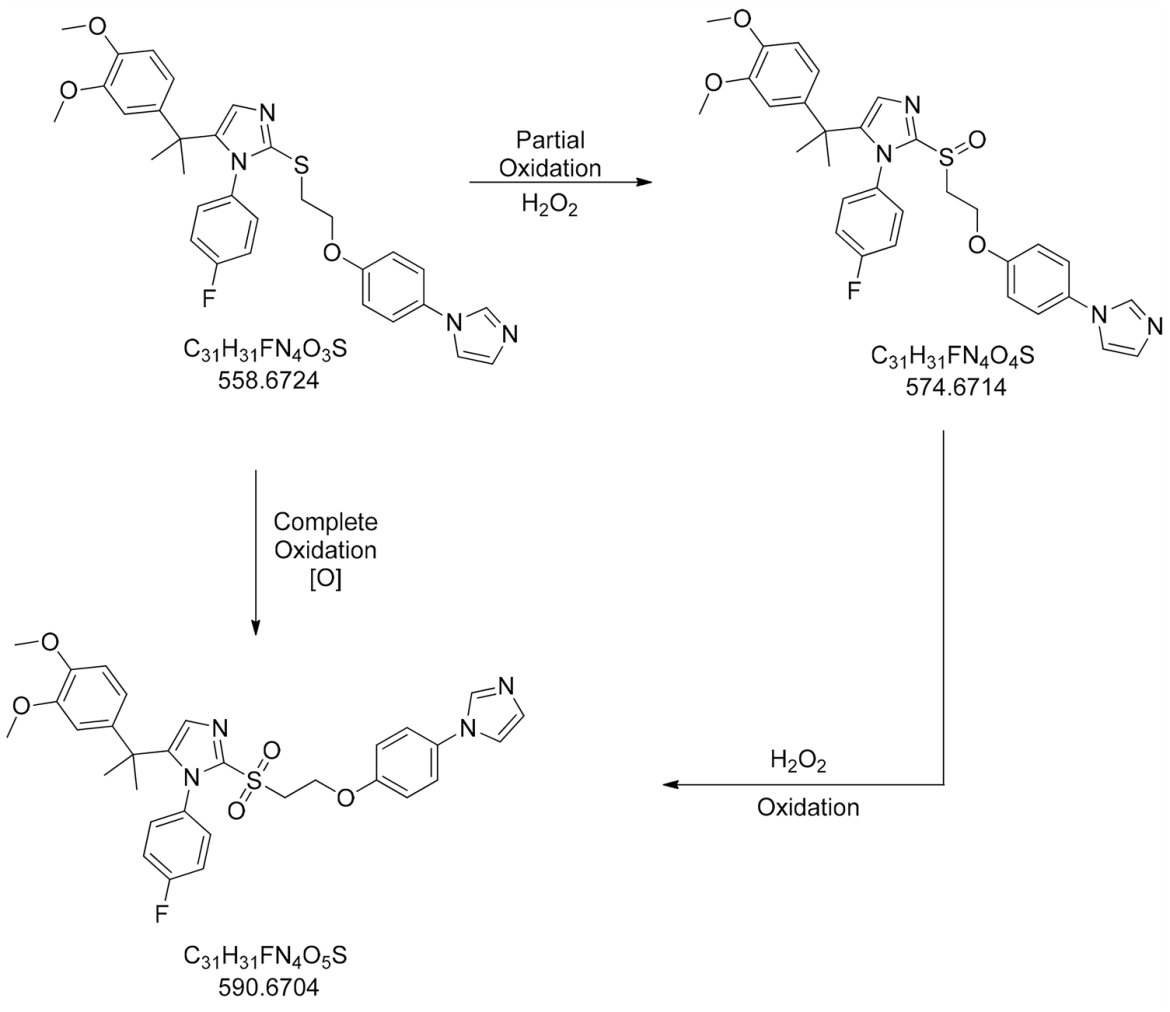
Oxidation degradation pathway of ZY12201

Form the above mentioned degradation study it was demonstrated tht in the presence of oxidizing agent ZY12201 has been degraded to its related substance. Thus, the degradation pathway has been confirmed with help of LC–MS system which is shown in Sect. 4.3. Figure 2 shows the degradation pathway of compound ZY12201 in presence of oxidizing agent like hydrogen peroxide.

## Conclusion

The developed liquid chromatography method is a rapid, precise, selective and capable for separation of ZY12201 from its degradation products with good resolution. Additioanlly, this method can be used for routine testing and stability analysis in quality control laboratories to check purity of ZY12201 in bulk and pharmaceutical formulation. The product must be packed in air tight container with nitrogen packing for stability studies and transportation purpose.

## Supplementary Information

Below is the link to the electronic supplementary material.

Supplementary file1 (PDF 691 kb)
