# Supplementary material for: Identification of degradation impurity of TGR5 receptor agonist-ZY12201 by LC–MS technique during force degradation study
Source: SN Appl Sci. 2021 May 25;3(6):660. doi: 10.1007/s42452-021-04660-y (PMC8144688; doi:10.1007/s42452-021-04660-y)

Supporting Information

Identification of degradation impurity of TGR5 receptor agonist - ZY12201 by LC-MS technique during force degradation study

Chandrakant Sojitra[a,b,c], Chintan Dholakia[a], Padmaja Sudhakar[b], Kumar K. Singh[a] and Sameer Agarwal[c,*]

[a]Cadila Healthcare Limited, API Division, Sarkhej-Bavla N.H. No. 8 A, Changodar, Ahmedabad – 382 210, India;
[b]Department of Chemistry, Faculty of Science, M.S.University of Baroda, Baroda – 390 002, India;
[c]Zydus Research Centre, Cadila Healthcare Ltd. Sarkhej-Bavla N.H. No. 8 A, Moraiya, Ahmedabad – 382 210, India;
*E-mail: sameeragarwal@zyduscadila.com

Contents

1. Chromatogram of control sample S2
2. Purity chromatogram of Acid treated sample S3
3. Purity chromatogram of Alkali treated sample S4
4. Purity chromatogram of Oxidation treated sample S5
5. Purity chromatogram of Photo degraded sample S6
6. Purity chromatogram of Thermally treated sample S7
7. Mass spectra of oxidation degraded sample S8

Corresponding Author

*E-mail: sameeragarwal@zyduscadila.com; or Sameer_ag@yahoo.com (SA); Fax: +91-2717-665355; Tel: +91-2717-665555

ORCID:

Sameer Agarwal: 0000-0002-7039-3028

Fig. S-1. Chromatogram of control sample

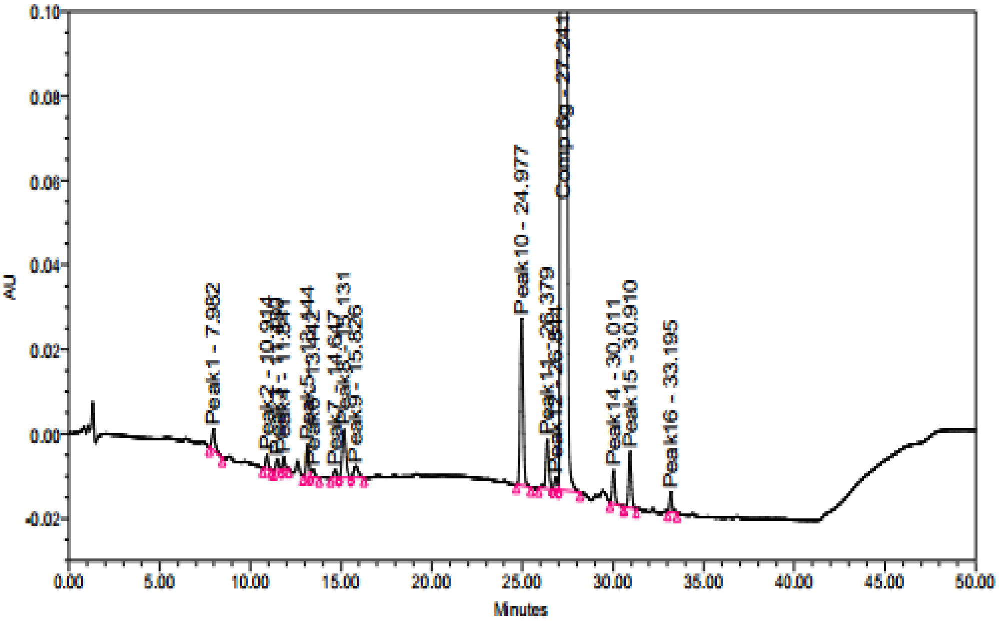

	Peak Name	RT	Area	% Area
1	Peak1	7.982	62345	0.15
2	Peak2	10.914	30197	0.07
3	Peak3	11.480	31595	0.08
4	Peak4	11.841	28468	0.07
5	Peak5	13.144	74555	0.18
6	Peak6	13.442	23405	0.06
7	Peak7	14.647	19275	0.05
8	Peak8	15.131	164861	0.40
9	Peak9	15.826	47547	0.12
10	Peak10	24.977	460236	1.13

	Peak Name	RT	Area	% Area
11	Peak11	26.379	136821	0.33
12	Peak12	26.844	33291	0.08
13	Comp 6g	27.241	39518165	96.62
14	Peak14	30.011	81132	0.20
15	Peak15	30.910	140015	0.34
16	Peak16	33.195	47697	0.12

Fig. S-2. Purity chromatogram of Acid treated sample

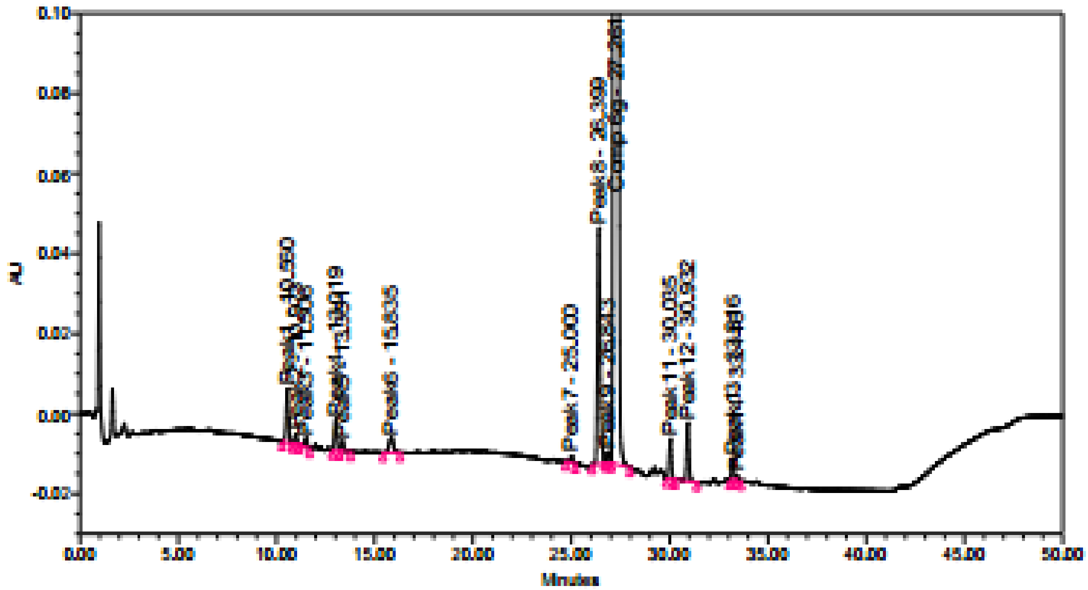

	Peak Name	RT	Area	% Area
1	Peak1	10.560	117287	0.37
2	Peak2	10.973	21188	0.07
3	Peak3	11.506	22414	0.07
4	Peak4	13.019	66794	0.21
5	Peak5	13.351	22363	0.07
6	Peak6	15.835	54024	0.17
7	Peak7	25.000	17011	0.05
8	Peak8	26.399	613550	1.95
9	Peak9	26.843	28196	0.09
10	Comp 6g	27.261	30292953	96.07

	Peak Name	RT	Area	% Area
11	Peak11	30.035	90459	0.29
12	Peak12	30.932	133626	0.42
13	Peak13	33.216	43084	0.14
14	Peak14	33.416	8908	0.03

Fig. S-3. Purity chromatogram of Alkali treated sample

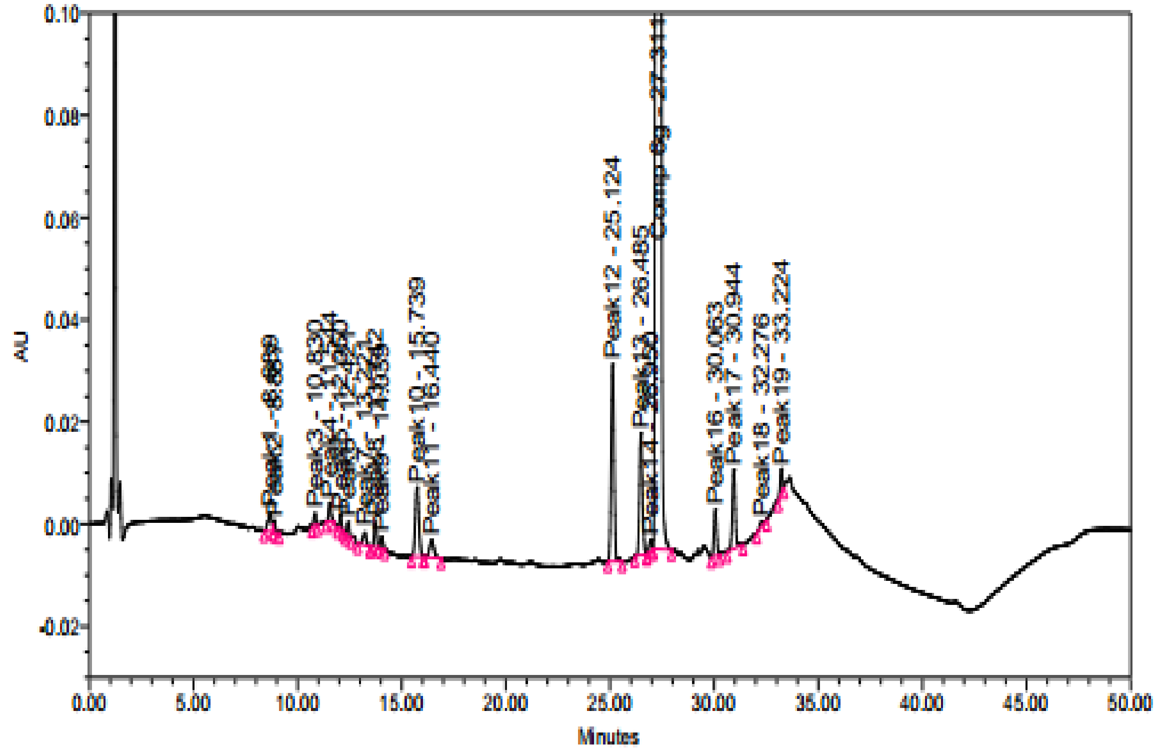

	Peak Name	RT	Area	% Area
1	Peak1	8.689	46530	0.15
2	Peak2	8.887	17224	0.05
3	Peak3	10.830	15407	0.05
4	Peak4	11.544	28176	0.09
5	Peak5	12.050	18398	0.06
6	Peak6	12.421	16553	0.05
7	Peak7	13.221	27529	0.09
8	Peak8	13.742	40992	0.13
9	Peak9	14.039	20106	0.06
10	Peak10	15.739	166976	0.53

	Peak Name	RT	Area	% Area
11	Peak11	16.440	50604	0.16
12	Peak12	25.124	380043	1.21
13	Peak13	26.485	240807	0.77
14	Peak14	26.950	20221	0.06
15	Comp 6g	27.311	30089561	95.65
16	Peak16	30.063	83150	0.26
17	Peak17	30.944	150397	0.48
18	Peak18	32.276	10185	0.03
19	Peak19	33.224	36455	0.12

Fig. S-4. Purity chromatogram of Oxidation treated sample

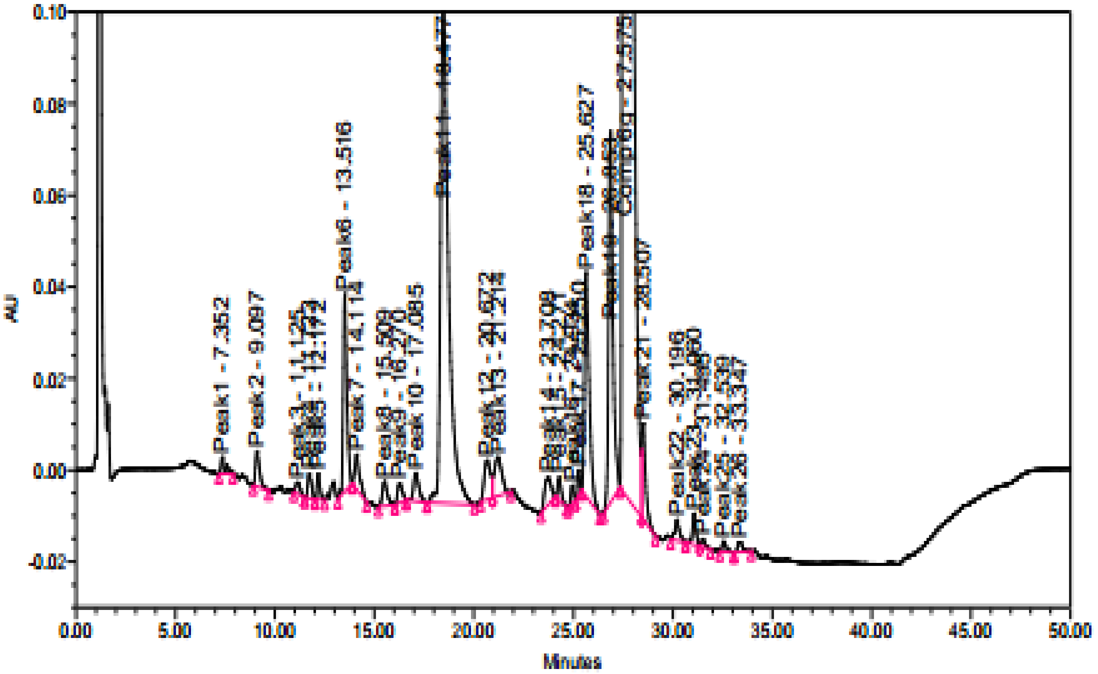

	Peak Name	RT	Area	% Area
1	Peak1	7.352	53659	0.15
2	Peak2	9.097	112682	0.31
3	Peak3	11.125	38292	0.11
4	Peak4	11.723	70451	0.19
5	Peak5	12.172	59753	0.16
6	Peak6	13.516	550438	1.52
7	Peak7	14.114	131948	0.36
8	Peak8	15.509	95036	0.26
9	Peak9	16.270	68518	0.19
10	Peak10	17.085	114631	0.32

	Peak Name	RT	Area	% Area
11	Peak11	18.477	3086675	8.50
12	Peak12	20.672	189782	0.52
13	Peak13	21.214	223653	0.62
14	Peak14	23.708	135305	0.37
15	Peak15	24.271	60708	0.17
16	Peak16	24.934	37361	0.10
17	Peak17	25.250	51415	0.14
18	Peak18	25.627	841549	2.32
19	Peak19	26.853	1203093	3.31
20	Comp 6g	27.575	28690747	78.98

Fig. S-5. Purity chromatogram of Photo degraded sample

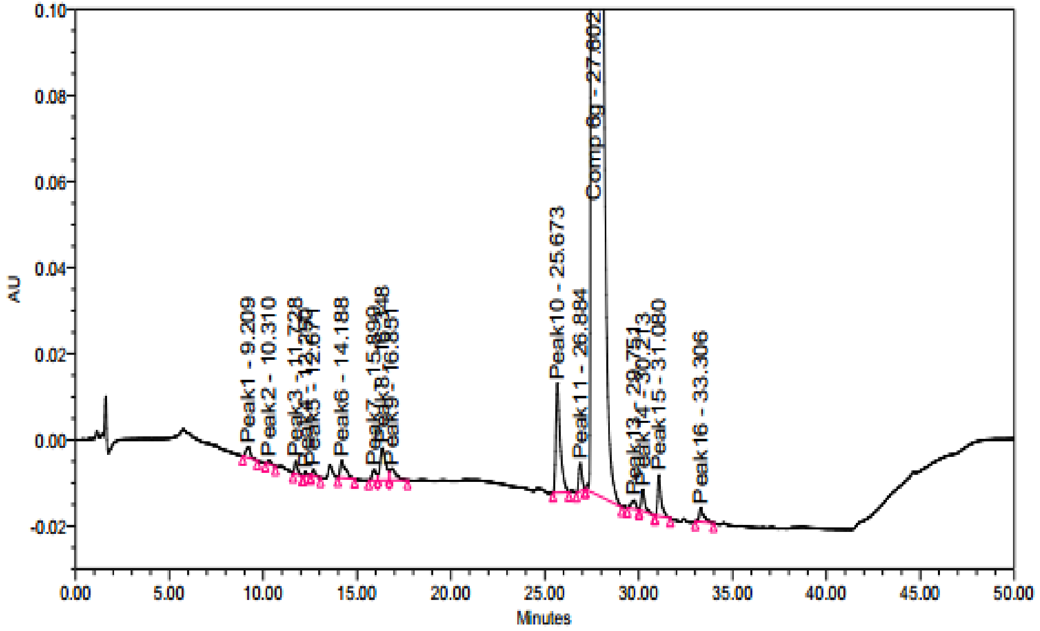

	Peak Name	RT	Area	% Area
1	Peak1	9.209	41749	0.10
2	Peak2	10.310	11590	0.03
3	Peak3	11.728	32544	0.08
4	Peak4	12.250	9235	0.02
5	Peak5	12.671	13125	0.03
6	Peak6	14.188	77288	0.18
7	Peak7	15.899	37720	0.09
8	Peak8	16.348	153794	0.36
9	Peak9	16.851	62999	0.15
10	Peak10	25.673	411739	0.96

	Peak Name	RT	Area	% Area
11	Peak11	26.884	75202	0.18
12	Comp 6g	27.602	41651845	97.18
13	Peak13	29.751	37873	0.09
14	Peak14	30.213	66037	0.15
15	Peak15	31.080	125030	0.29
16	Peak16	33.306	53728	0.13

Fig. S-6. Purity chromatogram of thermally treated sample

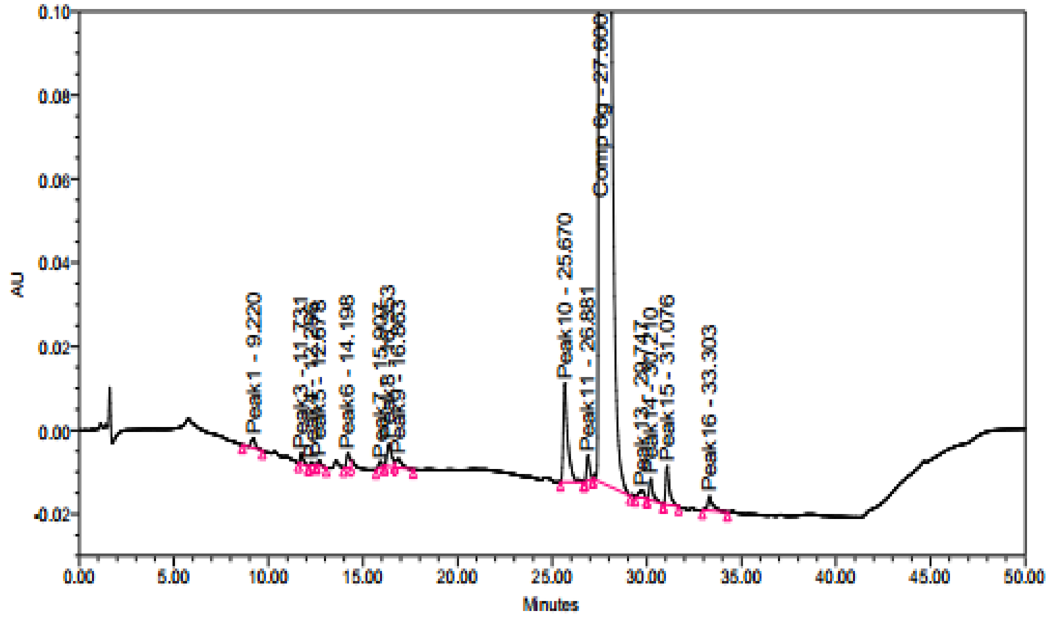

	Peak Name	RT	Area	% Area
1	Peak1	9.220	39979	0.10
2	Peak3	11.731	28271	0.07
3	Peak4	12.256	7213	0.02
4	Peak5	12.678	13208	0.03
5	Peak6	14.198	45158	0.11
6	Peak7	15.907	17231	0.04
7	Peak8	16.353	92434	0.23
8	Peak9	16.863	44445	0.11
9	Peak10	25.670	389471	0.96
10	Peak11	26.881	69205	0.17
11	Comp 6g	27.600	39483876	97.46
12	Peak13	29.747	32998	0.08
13	Peak14	30.210	65416	0.16
14	Peak15	31.076	119294	0.29
15	Peak16	33.303	63962	0.16

Fig. S-7. Mass spectra of oxidation degraded sample

2-Thio Imidazole Comp 6g_Redox Degradation_Optimised Method.lcd
Sample Name : 2-Thio Imidazole Comp 6g_Redox Degradation
Sample ID :
Acquired by : Riddham Patel
Date Acquired : 13/01/18 16:07
Vial# : 62
Injection Volume : 50
Method File : 2-Thio Imidazole Comp 6g_RS_Optimised_Q1_Prod_Column-2.lcm
Data File : D:\Mass Data\LC-MS\2018-January\13-01-18\2-Thio Imidazole Comp 6g_Redox Degradation_Opti

Chromatogram
2-Thio Imidazole Comp 6g_Redox Degradation_Optimised Method.lcd

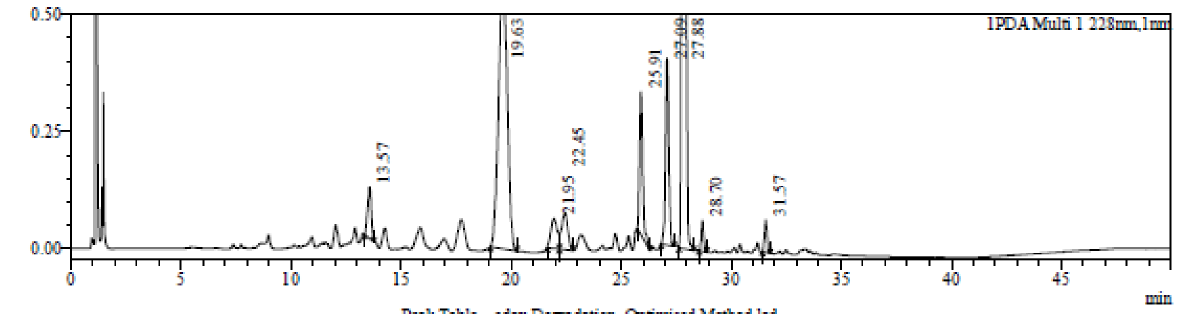

Peak Table ...edox Degradation_Optimised Method.lcd
PDA Ch1 228nm

Ret. Time	Area	Area%
13.57	1233578	1.66
19.63	15841237	21.33
21.95	1310873	1.76
22.45	1558639	2.10
25.91	3075984	4.14
27.09	4100328	5.52
27.88	46095745	62.06
28.70	523967	0.71
31.57	541283	0.73
	74281634	100.00

MS Spectrum
2-Thio Imidazole Comp 6g_Redox Degradation_Optimised Method.lcd

Line#:1 R.Time:---(Scan#:---)
MassPeaks:3
Spectrum Mode:Averaged 13.480-13.914(1614-1665) Base Peak:284.10(3733216)
BG Mode:Averaged 13.327-13.429(1596-1608) Segment 2 - Event 13

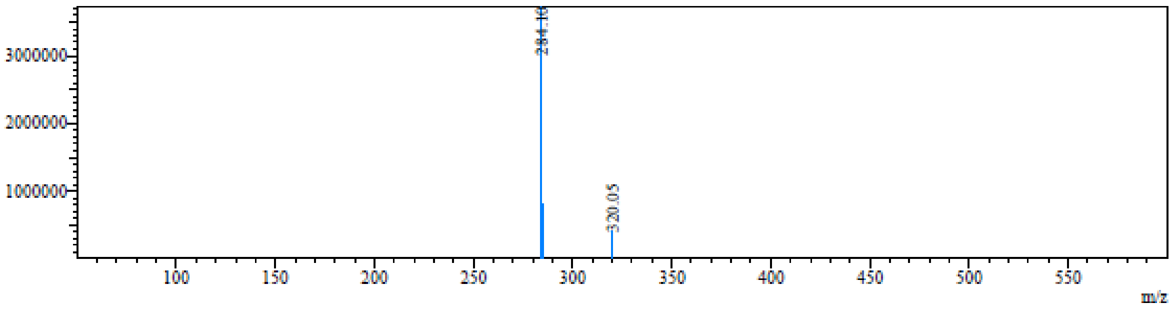

Spectrum Mode:Averaged 19.042-20.675(2277-2473) Base Peak:575.25(14341537)
BG Mode:Averaged 18.808-18.908(2249-2261) Segment 1 - Event 1

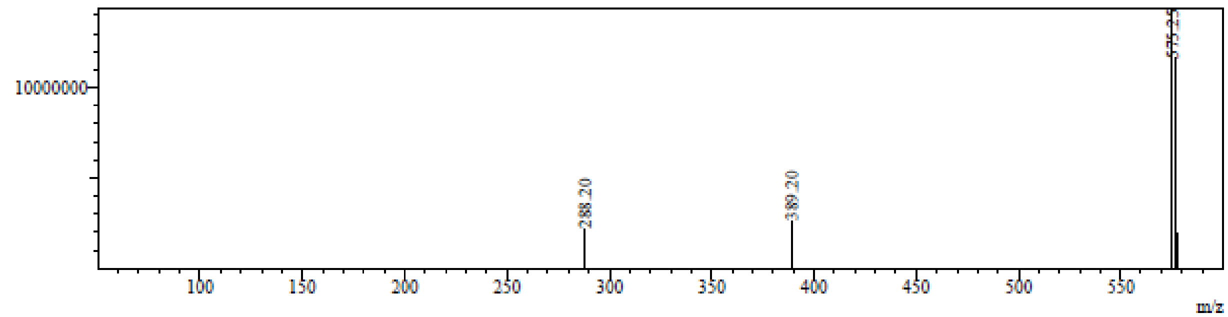

Spectrum Mode:Averaged 21.692-22.292(2595-2667) Base Peak:417.20(18445264)
BG Mode:Averaged 21.542-21.625(2577-2587) Segment 1 - Event 1

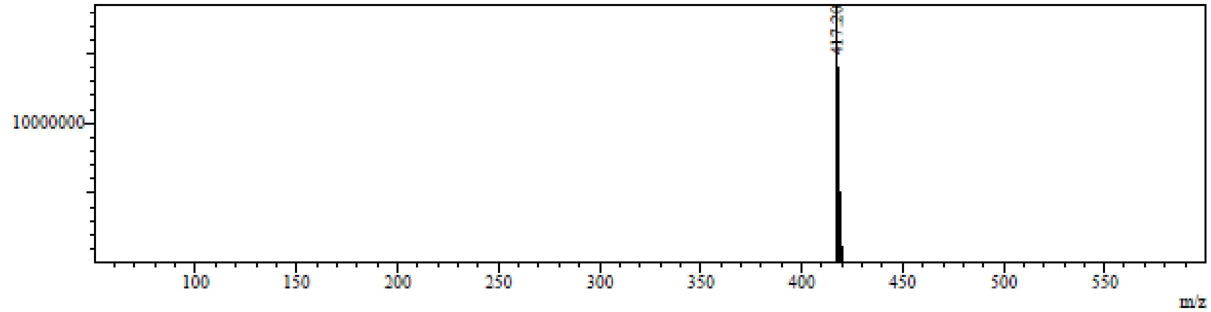

Spectrum Mode:Averaged 22.267-22.867(2664-2736) Base Peak:593.30(18046748)
BG Mode:Averaged 22.017-22.092(2634-2643) Segment 1 - Event 1

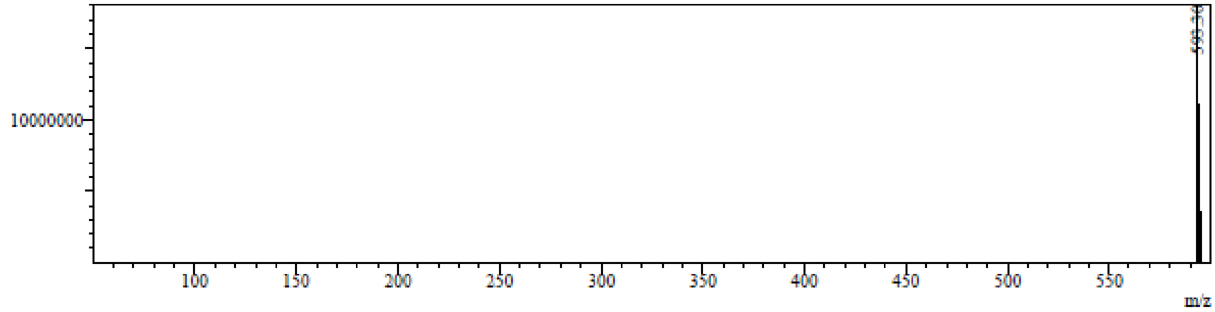

Spectrum Mode:Averaged 25.825-26.275(3091-3145) Base Peak:536.30(17636279)
BG Mode:Averaged 25.658-25.758(3071-3083) Segment 1 - Event 1

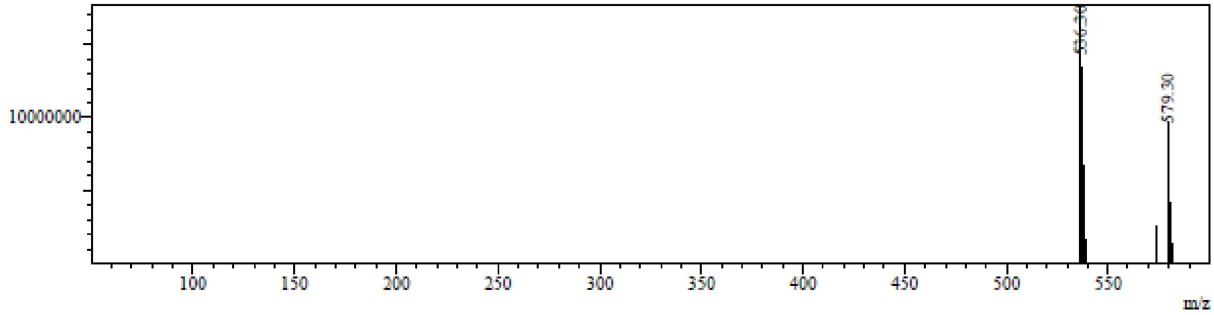

Spectrum Mode:Averaged 26.975-27.492(3229-3291) Base Peak:508.25(18200934)
BG Mode:Averaged 26.808-26.875(3209-3217) Segment 1 - Event 1

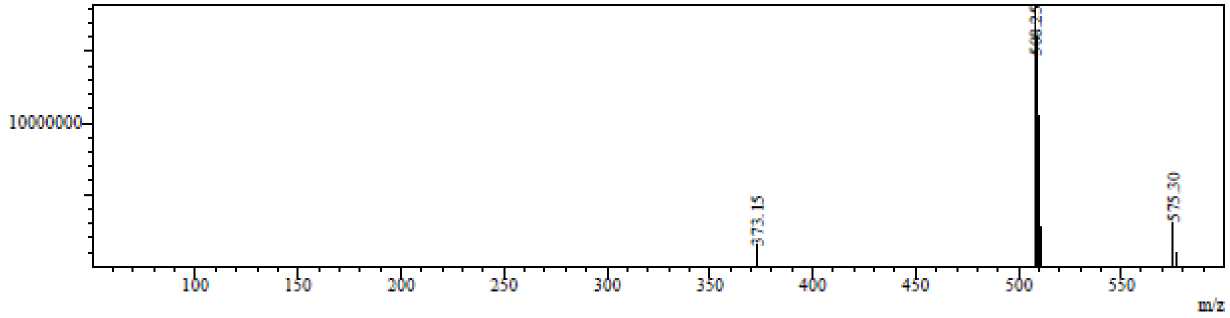

Spectrum Mode:Averaged 27.717-28.517(3318-3414) Base Peak:559.25(18985254)
BG Mode:Averaged 27.567-27.592(3300-3303) Segment 1 - Event 1

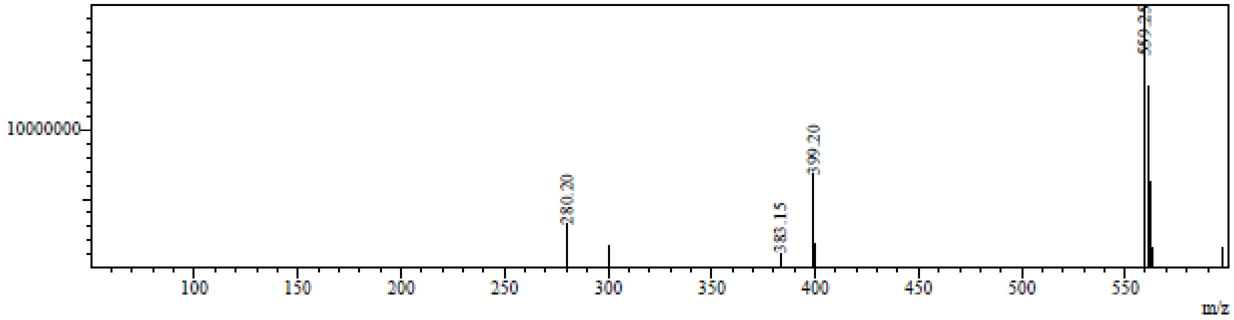

Spectrum Mode:Averaged 28.617-29.042(3426-3477) Base Peak:575.25(12714208)
BG Mode:Averaged 28.392-28.517(3399-3414) Segment 1 - Event 1

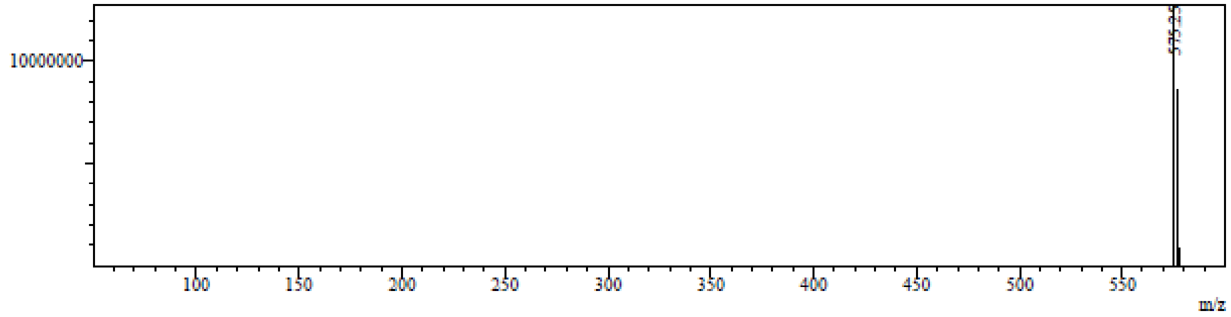

Spectrum Mode:Averaged 31.517-31.867(3774-3816) Base Peak:522.25(14070601)
BG Mode:Averaged 31.442-31.467(3765-3768) Segment 1 - Event 1

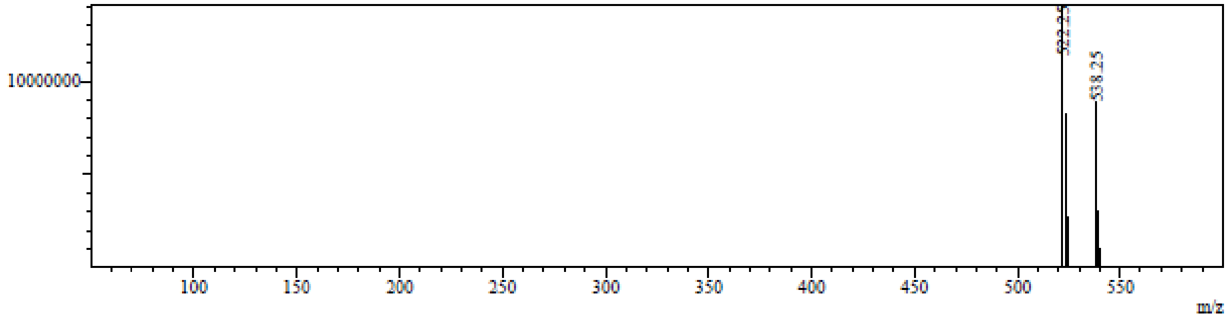

Supplement: Supplementary file 1 — Supplementary file1 (PDF 691 kb) [file 42452_2021_4660_MOESM1_ESM.pdf]
